# Multidimensional Disability Evaluation and Confirmatory Analysis of Older Adults in a Home-Based Community in China

**DOI:** 10.3389/fpubh.2022.899303

**Published:** 2022-06-21

**Authors:** Ying Han, Liangwen Zhang, Ya Fang

**Affiliations:** ^1^State Key Laboratory of Molecular Vaccinology and Molecular Diagnostics, School of Public Health, Xiamen University, Xiamen, China; ^2^School of Economics, Xiamen University, Xiamen, China

**Keywords:** disability, indicator system, Seemingly Unrelated Regression, Alkire and Foster approach, home-based care

## Abstract

A robust multi-dimensional disability assessment standard was constructed to consider physical condition, care resources, and social interaction that might lead to disability, to provide a basis for accurate identification of care needs for older people aged 60 and above in a home-based community. Based on the “Capability approach” theory, the Alkire-Foster method was applied to assess the multidimensional disability. This was followed by the confirmatory analysis of the Seemingly Unrelated Regression Estimation. Adjusted Bourguignon and Chakravarty index was also calculated to analyze the sensitivity to further support our conclusions. We constructed a multi-dimensional disability indicator system by combining physical condition, care resources, and social interaction. Findings presented that the impairment of individuals' motor ability, ability to manage disease, cognitive psychology, and communication skills and social interaction contributed to multidimensional disability the most. And older people who are female, aged over 65, with lower BMI, living in rural areas, with a lower education level, getting more formal care, and with relatively higher creatinine, tend to face a higher risk of deprivation in overall multidimensional disability. Therefore, the targeted interventions to improve health literacy, nutrition, skill of disease management, social networks, and communication skills for older people and also timely detection of the abnormal changes in potential biomarker indicators of them is necessary to delay disability and prevent its occurrence.

## Introduction

Disability in older people is a common problem and in most cases these disabilities are chronic conditions (1). The prevalence of disability varies among studies, depending on the criteria used, but generally increases with advancing age (2–4). According to a report from the World Health Organization (WHO) and the World Bank, over 1 billion people live with a form of disability (5). Likewise, these figures in China are estimated to increase with the aging of the population, with advanced aged and disability becoming a severe social problem (6). In 2021, the proportion of the population aged 60 years and older reached 0.26 billion (7), and is projected to reach 0.48 billion by 2025, with approximately 20% of disabled older adults (8). As the population ages and the family supporting function weakens, disability is becoming an increasingly important concept because of its adverse effects on health outcomes, health care costs, and quality of life (9). Furthermore, disability is a better predictor of adverse events in older adults, more predictive than multimorbidity or polypharmacy (10, 11), and thus should be looked at first for different disease approaches in older adult populations. Hence, the measurement of disability plays an increasingly important role in public health and research on the community home-based aging population.

It is clear that health measurements must be based on a specific conceptual framework; thus, the concept of disability must be clearly defined. The definition of disability differs according to what model of disability is considered. There are three relevant models-the medical, the social model, and the International Classification of Functioning, Disability and Health (ICF)- that lead to different conceptual frameworks of disability (12–14).

Indeed, the conceptual framework underlying the identification of what disability entails and its measurement has implications for the estimation of prevalence and research. The medical model conceived within the perspective of disability will focus on disadvantages of the individual considered as resulting from his/her impairment (15). In this model, the measurement of prevalence is based on evaluation of the number of persons within a series of categories of impairments, which are considered as limitations in health condition, across a range of basic functions and structures of the body. Within the social model, disability is considered to result from barriers erected by society that prevent the full participation in social and economic life (16). This model will not only focus on impairment but will include the identification of barriers within the social environment that create the disabling situation. In addition, the WHO developed ICF, which defines and measures disability as impairments in body function or structure (such as vision loss), that results in activity limitations (for instance difficulties in driving a car), and in restriction in individual participation in the context of their daily life (driving to work for instance) (17). According to the ICF, current research measured using a multidimensional approach in terms of disability, including self-care [activities of daily living (ADL) and instrumental activities of daily living (IADL)] (18), mobility (19), hearing (20), vision (21), cognitive function (19), pathology (20), blood chemistry (22), and disease status (22), frailty (23). However, all the three models presented above do not allow for in-depth considerations of personal factors, available resources, or social networks (24, 25).

We argue that most studies focus on certain factors, but without considering psychological indicators, individual situation, the ability of disease management, social behavior, care sources, and other important multidimensional disability indicators. In addition, there are factors that allow individuals to convert resources into capabilities. Those conversion factors could be internal and external. The internal conversion factors refer to physical conditions and personalities, which can reflect individual functioning. The external conversion factors included social and/or environmental characteristics (26). Over the last decade, however, there have been major steps taken to reconcile the various approaches by looking at the situation of individual interaction, collective resources, and disabling condition that may lead to individual impairment and a social disability.

The framework of Capability approach, elaborated by Amartya Sen and others, goes beyond such models, with the concept of disability being closely associated with deprivation of capabilities through the means of various personal characteristics, which included individual damage, available resources, and environmental conditions (27, 28). On the basis of such work, we made efforts to improve and standardize the measurement of disability in population-based surveys taken from the Capability approach. The use of the capability approach is rather an innovative way of assessing disabling situations (29).

In terms of methodological research, current studies on multidimensional capability deprivation focuses on poverty, and the assessment of multidimensional poverty content and methods have been relatively well developed and widely used (30). According to Amartya Sen's “approach capability” theoretical framework, poverty and disability have commonalities in multidimensional calculations and measurements, and both can be considered as a consequence of multidimensional capability deprivation. Therefore, this paper mainly draws on multidimensional poverty assessment methods for disability. There are widely used methods for measuring multidimensional poverty including the factor analysis and cluster analysis (31), the axiomatic approach (32), the information theory approach (33), multiplicative FGT index and the classes of indices (34), and AF methods (35). Among them, the AF method, also known as the “double limit method,” was based on Amartya Sen's “capability poverty theory” and was developed by Alkire and Foster, and that lead to the construction and decomposition of the multidimensional poverty index (36). Alkire and Foster designed the multidimensional poverty measures, which has been applied in practice, and their scientific validity was demonstrated in monitoring poverty, focusing on poor people, and coordinating poverty reduction efforts in the real world (37). The AF method is a multidimensional poverty measurement method adopted by the United Nations Development Programme (UNDP). It is widely used in poverty studies around the world since Dhongde and Haveman have adjusted the dimensions of poverty deprivation and indicator thresholds (38–40).

Dhongde and Xu argued that most researchers have some controversial assumptions in the process of calculating multidimensional deprivation measurement (41). It is usually assumed that each of the attributes or dimensions under consideration is cardinally measurable along a real interval. In fact, in studies measuring various types of material deprivation (i.e., material deprivation index), there are difficulties such as unavailability or difficulty in collecting expenditure data, and only binary information can be relied on to assess poverty deprivation (42). In addition, when assessing health status, information can be obtained by measuring indicator dimensions such as weight, frequency of illness, blood pressure, blood glucose, and cholesterol levels. However, it is extremely difficult to provide a cardinal measurement of an individual's frailty and disability, since cardinal measurements are demanding and its data are difficult to obtain. Moreover, the important feature of the variables for poverty assessments is that they are discrete in nature and the attributes of these different types of dimensions do not exist separately and can be alternated. Thus, poverty measures based on continuous variables are not suitable in this setting and the assumption of a discrete domain is mandatory (43). With respect to the individual deprivation dimension hypothesis, for each attribute or dimension, two levels of deprivation are mostly used: the individual dimension is deprived (dimensional assignment of 1) or not deprived (assignment of 0) (44). Although this binary, ordered measure of individual deprivation across dimensions is not as informative as an ordered measure involving more than two deprivations, there is a broad consensus on the information it yields. The United Nations Multidimensional Poverty Index (UNMPI), published annually for more than 100 countries, makes use of information from binary variables. Indeed, binary variables are central to the counting methodology (45).

Using this simple binary measurement for each attribute of individual deprivation, we derive the overall multidimensional deprivation of an individual to be a weighted average deprivation attribute with increased functionality (41). This creates a discontinuity in the measurement of poverty that may penalize welfare equality policies and development processes. This situation is inevitably exacerbated when multidimensional discontinuities are introduced across multiple dimensions. This source can be corrected by using the more general MPI found in AF indices. For example, when α > 0, the AF exponent is implied. In addition, it seems some attributes of fundamental importance for individuals' health should be differentiated from other attributes of less importance (46). In this regard, an increase from 0 to l deprivation in terms of an attribute of the former type cannot be offset by a decrease from 1 to 0 in deprivation in other types of attributes. Under such a premise, there is no notion of a slight increase in personal deprivation in attributes. This indicates that the MPI might fail to meet the properties that one would expect multidimensional poverty indices to obey: continuity and monotonicity. However, robustness techniques and sensitivity can be used to address some of the shortcomings of the use of such indices (47).

Given this, Bourguignon and Chakravarty proposed an adjusted multidimensional poverty index (32). The following two features are included: (1) individuals should be considered poor if they cannot meet the basic level in at least one of the poverty-related dimensions; (2) individuals with the same level of poverty in some dimensions but with different degree of insufficiency in other aspects should be different. Based on these premises, an adjusted poverty index, called BC^a^ index, was widely applied to measure poverty in the OECD countries (48).

In this paper, literature analysis and Alkire-Foster method were used to explore the scope of the overall older capacities to perform a multi-dimensional disability evaluation index system applicable to a home-based community in China. And also, a confirmatory analysis was conducted by the Seemingly Unrelated Regression Estimation and adjusted Bourguignon and Chakravarty index (BC^a^). The establishment of the multidimensional disability index system contributes to the formation of a standardized national assessment operation process (SOP) for the disabled older adults in a home-based community, and the standardization and equity of the national implementation. According to the multi-dimensional disability status of older adults, we can match the corresponding care level for training the professional nursing staff, which is helpful for the rational allocation of long-term care resources for the disabled older population, and eventually provide a basis for improving the long-term care system for disabled older adults.

## Methods

### Study Population

We used data from the first wave of the China Health and Retirement Longitudinal Study (CHARLS) (49). The CHARLS is a population-based cohort study among adults aged 45 and older from 28 provinces and municipalities of China. Details on the methods and sampling have been published previously (50). Within a total sample size of 17,708 participants aged 45 and older from the 2011 baseline data, a sum of 9,940 individuals (aged less than 60) and 1,776 individuals (with missing variables, incomplete information, and living in institutionalized care) were excluded. The final sample of 5,992 individuals aged 60 years and older living in a home community was included in the study. Based on data availability, relevant indicators, and dimensions from systematic literature analysis, our conceptual framework of disability of older adults was defined in terms of three aspects of individuals, resources, and society, and mainly covers issues on sociodemographic characteristics, family, behavior, cognitive, psychological, environmental, and biological factors that affect health and longevity:

(1) Sociodemographic characteristics: age, sex, place of residence (urban or rural), marital status, per-capital annual household income, educational level.
(2) Daily behavior: social activities, motor competence.
(3) Health behavior: sleep duration, formal (paid) and informal (unpaid) care.
(4) Biological factors: blood pressure, BMI, white blood cell count, platelet count, blood creatinine, glycosylated hemoglobin, total cholesterol.
(5) Multidimensional disability related variables.

### Defining the Multi-Dimensional Disability

(1) Construct multi-dimensional Disability Index (MDI)

A-F method was used to construct MDI; the matrix X = [*x*_*ij*_] defines the *n*×*m* dimension of *i*^*th*^ individuals across *j* variables, which was set to reflect the deprivation of individuals, where *n* represents the total number of individuals and *m* represents the total number of dimensions. The first step is to identify the multidimensional disability of individuals:


(1) $${\text{α}}_{ij}=\{\begin{array}{c} \omega_{j}{\left(\frac{z_{j}-x_{ij}}{z_{j}}\right)}^{\alpha },\;x_{ij}<z_{j}\\ \text{0},\;x_{ij}>z_{j}\; \end{array}$$

In formula (1), under dimension j, the weight was defined as ω_*j*_ (ω_*j*_>0), which is the weight vector of (1 × *j*). *z*_*j*_ represented the first critical value of the set indicator m and *z* is the disability threshold vector of (1 × *j*). If the individual's dimension of deprivation *x*_*ij*_>*z*_*j*_, then $g_{ij}^{0}=1$, otherwise $g_{ij}^{0}=0$, g' represented the deprivation matrix of (*n*×*d*). That is, $g_{ij}^{0}$ (α = 0) was used to determine whether individual *i* is deprived under dimension *j*: deprived (g = 1), and Non-deprived (g = 0).

The second step is to determine whether the individual is in a multidimensional state of disability by assuming that the individual *i* has a deprivation score of all dimensions $c_{i}=\overset{m}{\underset{j=1}{\sum }}g_{ij}^{0};$ given the critical value *k* (the second critical value) and the disability recognition function for individual *i*: ρ_*k*_(*X*_*i*_, *Z*). Specifically: individual i was defined as disabled when *c*_*i*_>*k*, ρ_*k*_(*X*_*i*_, *Z*) = 1; while non-disability if *c*_*i*_<*k*, ρ_*k*_(*X*_*i*_, *Z*) = 0.

All individuals *n* in multidimensional disability could be recognized via the two steps above; the prevalence of multidimensional disability is expressed as $H=\frac{q}{n}$, where *q* is the total of multidimensional disability. However, considering *H* is insensitive to the changes of disability dimensions, corrections for deprived of matrix were performed by individual recognition function ρ_*k*_(*X*_*i*_, *Z*), yielding $g_{ij}^{\alpha }\left(k\right)=g_{ij}^{\alpha }\times \rho_{k}\left(X_{i},Z\right)$, and Modified deprivation score $c_{i}\left(k\right)=\overset{m}{\underset{j=1}{\sum }}g_{ij}^{0}\left(k\right).\;$Corrected incidence of disability (*M*_α_) was calculated from the equation:


(2) $$M_{\alpha }\;=P\left(\alpha ,X,Z\right)=\frac{1}{n}\Sigma_{i=1}^{q}\Sigma_{j=1}^{m}{\left(\frac{z_{j}-x_{ij}}{z_{j}}\right)}^{\alpha }\rho_{k}\left(X_{i},Z\right)$$

(3) $$\begin{array}{rcl} A & = & \Sigma_{i=1}^{q}\frac{c_{i}\left(k\right)}{q}=\Sigma_{i=1}^{q}\Sigma_{j=1}^{m}g_{ij}^{0}\left(k\right) \end{array}$$

(4) $$\begin{array}{rcl} H & = & q/n \end{array}$$

In estimating a multidimensional disability index using A-F framework, the disability rate (H) was multiplied with the average intensity of disability deprivation (*A*), *the fomula*:*M*_0_ = *H*×*A* reflects the proportion of the total number of deprivation dimensions experienced by all individuals to the total number of dimensions.

(2) Multidimensional decomposition

The multidimensional disability index constructed using the A-F method had decomposability and subgroup decomposability. *P*(α, *X*.*z*_*j*_) defines the disability decomposition across *j* variables, combined with equation (2), the disability index of each dimension and the contribution rate of each indicator to multidimensional disability can be calculated:


(5) $$\begin{array}{rcl} M_{\alpha j} & = & P\left(\alpha ,X,z_{j}\right)=\frac{1}{n}\Sigma_{i=1}^{n}\omega_{j}{\left(\frac{z_{j}-x_{ij}}{z_{j}}\right)}^{\alpha }\rho_{k}\left(X_{i},Z\right) \end{array}$$

(6) $$\begin{array}{rcl} P_{\alpha j} & = & \frac{M_{\alpha j}}{M_{\alpha }}=P\left(\alpha ,X,z_{j}\right)\;/\;P\;\left(\alpha ,X,Z\right) \end{array}$$

(3) Bourguignon and Chakravarty bidimensional index (BC^a^)

A new multidimensional poverty index, called Adjusted Bourguignon Chakravarty index, BC^a^, allows for adjustments by the attributes of disabled people who do not fall below the corresponding threshold level, without changing the identification of an individual as disability. Compared to other methods, the strength and the mechanism of this can be seen in Supplementary Table S2. The indexes were calculated by stata version 16.0 DASP package “imdp_bci.”

### Variables

The scope of the overall older capacities deprivation was calculated to construct a multi-dimensional disability evaluation index system applicable to the home-based community of China. There were three aspects (individuals, resources, and society) in terms of those indexes: individual level including self-care, motor ability, ability of processing diseases, cognitive mental status, and communication skills, and medical conditions. Self-care disability was defined when there existed at least one index negative, where the score indicated reduced ability to perform ADL, IALD, and frailty. Motor ability included balance, falls, postural transition, arm stretching, and mobility, while this capability impairment was evaluated by failing to complete one of them. Older people who sought medical treatment, exceptional treatment, or used assistive device, never adopt self-treatment as a loss in the ability of processing disease. Cognitive mental status and communication skills were experience-dependent, and encompasses cognition, memory, vision, hearing, sadness and depression condition, as this ability impairment was defined to exist with at least one declined capability. Among them, depression was measured by the Center for Epidemiologic Studies Depression Scale (CES-D) (51). The measurement of medical condition was derived from medical records where chronic diseases and physical disability are registered. Care resources constituted of access to caregivers and living arrangement. Older people who lived alone and far away from their children or lacked caregivers were defined as sources of care deficiency. Home settings as an environmental characteristic could be converted into capabilities, while disability of it was defined according to accessibility. Social interactions impairment results in activity limitations like restriction in interacting with friends, joining community clubs and community-related organizations, and failure to help friends. A summary statistic of the variables applied for this study are presented in Supplementary Table S1.

### Deprivation

The dependent variable is in each deprivation dimension of older people, and the independent variables are the influencing factors of the multidimensional disability. OLS model was adopted to analyze the factors affecting overall dimensional disability. However, the eight regression equations influenced by basic characteristics were constructed by:

(*Multi*_*daily* = *X*_1_*Y*_1_+ε_1_; *Multi*_*sports* = *X*_2_*Y*_2_+
ε_2;_*Multi*_*dealing* = *X*_3_*Y*_3_+ε_3_; *Multi*_*cognition* = *X*_4_*Y*_4_+ε_4;_*Multi*_*disease* = *X*_5_*Y*_5_+
ε_5_; *Multi*_*resources* = *X*_6_*Y*_6_+ε_6_; *Multi*_*living* = *X*_7_*Y*_7_ + ε_7_; *Multi*_*interaction* =
*X*_8_*Y*_8_ + ε_8_), regression equations above were superimposed to a Seemingly Unrelated Regression Estimation, SURE.

### Statistical Analysis

Statistical data processing was performed using stata version 16.0. A list of multidimension disability indexes was generated from literature review and panel discussion. These were initially classified into 3-level indexes including eight dimensions and 29 indexes. Each of the indexes was used to construct an MDI system by A-F method. Seemingly Unrelated Regression Estimation (SURE) was applied to analyze the influencing factors of deprivation of 8 dimensions in stata version 16.0.

## Results

### Constructed a Multi-Dimensional Disability Index for Home-Based Community Older Adults

#### Measurement of Disability in Each Dimension and Index

We first analyzed the disability of each index and dimension. Table 1 showed that 21 indexes had a greater than 20% disability prevalence, of which ADLs limited accounted for 23.78%, while IADLs limited prevalence was 5% higher than it. This results from the higher severity of dysfunction than ADLs, like the ability to take public transportation. However, the results also suggested that 29.47% of unattended older adults are more likely to have dysfunction. It is urgent to increase social and family support among this older population by strengthening existing long-term care (LTC) and social service decisions or creating new ones.

**Table 1:** Dimensions and indicators of the multidimensional disability and its cut-off.

**Dimensions-indicators**	**Deprivation cutoff**	**Weight**	**Deprived on dimensions and indicators (%)**
Self-care	1 = At least one impairment	1/8	38.98
ADL	1 = Dependently to complete ADLs	1/24	23.78
IADL	1 = Dependently to complete IADLs	1/24	28.73
Frailty	1 = Yes	1/24	4.42
Motor ability	1 = At least one impairment	1/8	72.41
Balance	1 = No	1/40	35.67
Falls	1 = Yes	1/40	19.59
Postural transition	1 = No	1/40	33.77
Arm stretching	1 = No	1/40	12.83
Mobility	1 = No	1/40	50.83
Ability of processing diseases	1 = At least one impairment	1/8	65.37
Receiving medical services	1 = Yes	1/32	21.76
Exceptional treatment	1 = Yes	1/32	11.72
Self-treatment	1 = No	1/32	48.60
Assistive Device	1 = Yes	1/32	9.75
Cognitive mental status and communication	1 = At least one impairment	1/8	76.49
Cognition	1 = Low cognition	1/48	40.47
Memory	1 = Poor	1/48	40.37
Vision	1 = Bad	1/48	40.25
Hearing	1 = Poor	1/48	20.14
Sadness	1 = Poor	1/48	1.60
Depression	1 = Yes	1/48	34.48
Medical condition	1 = At least one impairment	1/8	43.44
Chronic disease	1 = Yes	1/16	24.83
Physical disabilities	1 = Yes	1/16	22.98
Sources of care	1 = At least one impairment	1/8	39.75
Access of caregiver	1 = No	1/16	29.57
Living arrangement	1 = Live alone and far away from children	1/16	15.84
Home settings	1 = At least one impairment	1/8	28.92
Elevator in a four-story apartment	1 = No	1/16	3.92
Accessibility	1 = No	1/16	25.08
Social interactions	1 = At least three impairments	1/8	87.05
Interacted with friends	1 = No	1/40	65.92
Community club	1 = No	1/40	83.98
Provide help to friends	1 = No	1/40	95.58
Outdoor activities	1 = No	1/40	94.08
Community-related organization	1 = No	1/40	98.65

*1, disability or impairment in this index; 0, without any impairment in this index*.

#### The Evaluation of MDI

**Figure 1:**
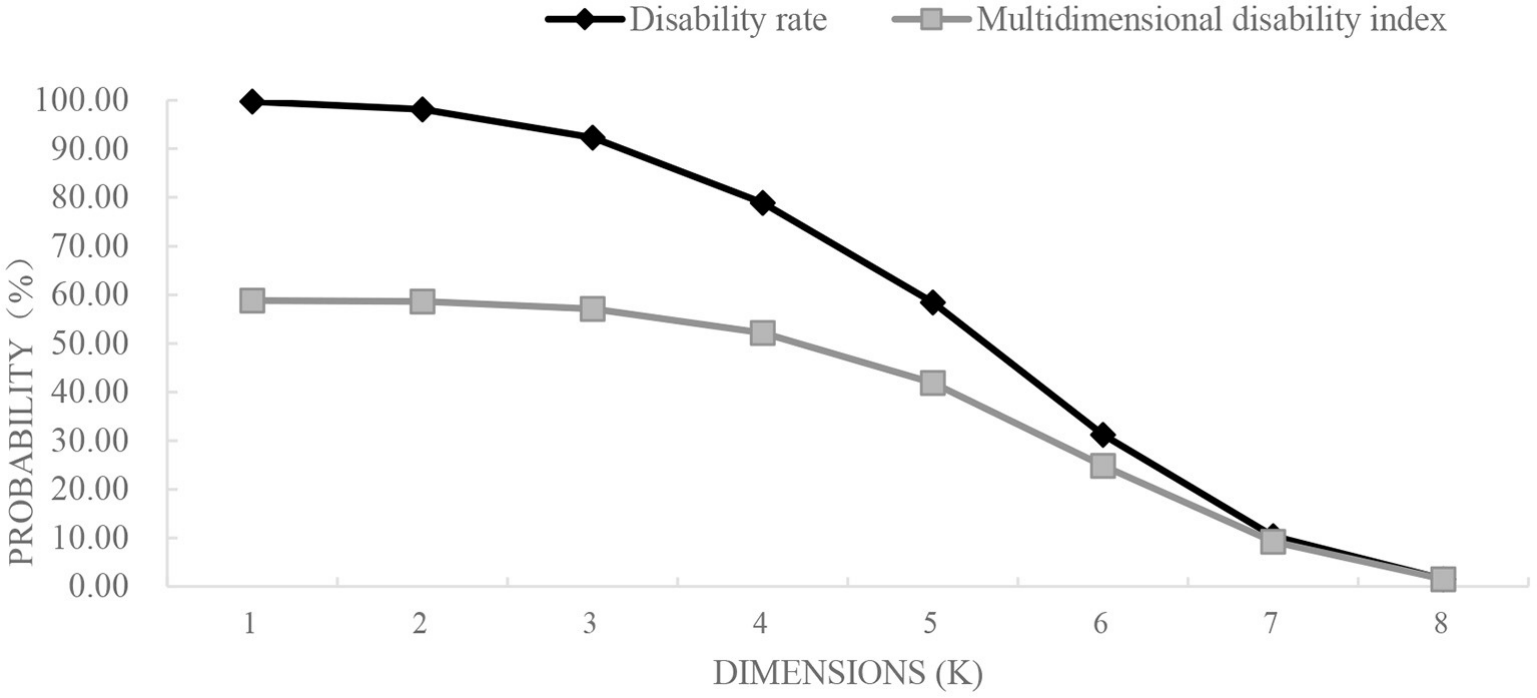
H and M_0_ occupancy relationship. H, disability rate; M0, multidimensional disability rate.

The multidimensional disability rate under different *k* values (*k* = 1, 2, …, 8), were shown in Figure 1 and Table 2. Older adults with disability accounted for 56.54% when *k* is equal to 1, while at *k* = 2, there presented 54.99% of older adults who had two or more dimensions of disability, and their multidimensional disability indexes were similar, indicating that most older adults had multidimensional disability. When *k* = 6, the proportion of multi-dimensional disability in older adults was 21.23%, which was close to the international common understanding that disability was the loss of functions such as bathing and toilet. The proportion of older adults with multidimensional disability was 7.52% when *k* = 7, and only 1.29% of older adults had eight dimensions of disability. Therefore, almost all of older people have 2 ~ 6 dimensions of disability, indicating that there are certain limitations of using ADL scale only to estimate disability.

**Table 2:** M_0_ value at different dimensions.

***k***	**Disability rate/H%**	**Average incapacitation/A%**	**Multidimensional disability index/M_**0**_%**	***k***	**Disability rate H/%**	**Average incapacitation /A%**	**Multidimensional disability index /M_**0**_%**
1	99.69	56.73	56.55	5	52.84	71.11	37.57
2	97.65	57.66	56.31	6	26.69	79.54	21.23
3	90.62	60.19	54.54	7	8.41	89.41	7.52
4	75.33	64.80	48.81	8	1.29	100	1.29

#### Analysis of Dimension Contribution Rate

Table 3 presented MDI that was applied to measure the contribution of each dimension to disability through data decomposition. Different dimensions(*k* = 1,2,…,8)for disability contribution are shown in Supplementary Figure S1. Results showed that 56.32% of the disabled population had two or more dimensions of impairment when *k* = 2. By decomposition, social interactions (19.12%), cognitive mental status and communication (16.91%), motor ability (16.01%), and ability of processing diseases (14.45%) had the highest contribution to disability, which was consistent with the value of dimension k (*k* = 3, 4, 5, 6, 7, 8).

**Table 3:** The contribution of multidimensional disability indexes (M_0_).

		**The contribution rate (%) of M**_**0**_							
**Cut-off**	**M_**0**_**	**Self-care**	**Cognition** **and communication**	**Ability of processing** **diseases**	**Motor ability**	**Medical condition**	**Sources of care**	**Home settings**	**Social interactions**
*k* = 1	56.55	8.62	16.91	14.45	16.01	9.60	8.79	6.39	19.24
*k* = 2	56.31	8.65	16.94	14.45	16.03	9.62	8.79	6.39	19.12
*k* = 3	54.54	8.92	16.98	14.33	16.12	9.68	8.85	6.40	18.71
*k* = 4	48.81	9.65	16.99	14.19	16.11	9.77	9.02	6.42	17.84
*k* = 5	37.57	11.04	16.43	13.88	15.93	10.24	9.28	6.48	16.73
*k* = 6	21.23	12.24	15.38	13.74	15.21	10.97	010.17	7.06	15.24
*k* = 7	7.52	12.77	13.96	13.18	13.85	12.46	11.30	8.71	13.77
*k* = 8	1.29	12.50	12.50	12.50	12.50	12.50	12.50	12.50	12.50

#### Comparison of Contribution Rate Among Gender, Age, and Living Places

Older people living in home-based communities, who were female, living in rural areas, and older than 65 years have higher multidimensional disability contributions (shown in Tables 4–6).

**Table 4:** Multidimensional disability index (M_0_) and contribution rate were broken down by gender under different *k* values.

***k***	**M**_**0**_			**Contribution**	
	**Total**	**Male**	**Female**	**Male**	**Female**
1	0.5654	0.2728	0.2930	0.4809	0.5191
2	0.5631	0.2704	0.2927	0.4802	0.5198
3	0.5454	0.2594	0.2860	0.4757	0.5243
4	0.4881	0.2261	0.2621	0.4632	0.5368
5	0.3757	0.1682	0.2075	0.4478	0.5522
6	0.2123	0.0953	0.1170	0.4491	0.5509
7	0.0752	0.0343	0.0409	0.4568	0.5432
8	0.0129	0.0060	0.0069	0.4691	0.5309

**Table 5:** Multidimensional disability index (M_0_) and contribution rate under different *k* values were broken down by age.

***k***	**M**_**0**_			**Contribution**	
	**Total**	**Less than 65**	**Oder than 65**	**Less than 65**	**Older than 65**
1	0.5655	0.207479	0.357927	0.366956	0.6330444
2	0.5631	0.206219	0.356883	0.36622	0.6337804
3	0.5454	0.197621	0.347792	0.362333	0.6376673
4	0.4881	0.171929	0.316239	0.352192	0.6478079
5	0.3757	0.127969	0.247761	0.340589	0.6594114
6	0.2123	0.065703	0.146595	0.309484	0.690516
7	0.0752	0.023257	0.051937	0.309293	0.6907074
8	0.0129	0.005026	0.007874	0.38961	0.6103896

**Table 6:** Multidimensional disability index (M_0_) and contribution rate under different *k* values were broken down by urban and rural areas.

***k***	**M**_**0**_			**Contribution**	
	**Total**	**Urban**	**Rural**	**Urban**	**Rural**
1	0.5655	0.104594	0.460812	0.475867	0.5241325
2	0.5631	0.10385	0.459252	0.474932	0.525068
3	0.5454	0.097945	0.447469	0.466822	0.5331776
4	0.4881	0.083065	0.405103	0.450605	0.5493946
5	0.3757	0.058039	0.317691	0.422219	0.5777812
6	0.2123	0.029774	0.182524	0.394853	0.6051467
7	0.0752	0.008614	0.066579	0.34104	0.6589595
8	0.0129	0.001173	0.011727	0.285714	0.7142857

#### Robustness Analysis Through Threshold Adjustment

Indicator threshold was used to measure the degree of deprivation of specific indicators of each disabled individual. If any small change in threshold will lead to a large change in multidimensional disability index, the selection of indicators and thresholds should be further checked (52). In order to test the sensitivity of MDI constructed by A-F method, the paper redefines the index threshold, and defines each failure index under a more strict condition while the dimensional threshold remains unchanged. That is, the failure of this indicator was defined as the highest level of impairment. For the lower limb ability, upper limb ability, and mobility, and ADLs and IDALs, the original “with difficulty but able to complete” and “with difficulty and need help,” were defined as the function limits of these indicators. After adjusting the threshold, the standard was changed into “unable to complete.”

The study found that, when Multidimensional disability assessment was carried out with the strict selection of index thresholds with other condition constants, the estimates of disability rate fell. When *k* = 5, older people with 2 or more dimensions of disability fell from 28.94 to 37.57%. However, four dimensions, namely social interactions, cognitive mental status and communication, motor ability, and the ability to process disease, still maintained the highest contribution rate of multidimensional disability among other dimensions, which was relatively consistent with the results found above. To sum up, the calculation result of adjusted indicator threshold is more robust compared with the evaluation result above (Tables 7, 8).

**Table 7:** Dimensions and indicators of the multidimensional disability and its cut-off after adjustment.

**Dimensions-indicators**	**Deprivation cutoff**	**Weight**	**Deprived on dimensions and indicators (%)**
Self-care	1 = At least one impairment	1/8	22.96
ADL	1 = Dependently to complete ADLs	1/24	8.08
IADL	1 = Dependently to complete IADLs	1/24	17.94
Frailty	1 = Yes	1/24	4.42
Motor ability	1 = At least one impairment	1/8	52.20
Balance	1 = No	1/40	35.67
Falls	1 = Yes	1/40	19.59
Postural transition	1 = No	1/40	1.62
Arm stretching	1 = No	1/40	4.96
Mobility	1 = No	1/40	16.81
Ability of processing diseases	1 = At least one impairment	1/8	65.37
Receiving medical services	1 = Yes	1/32	21.76
Exceptional treatment	1 = Yes	1/32	11.72
Self-treatment	1 = No	1/32	48.60
Assistive Device	1 = Yes	1/32	9.75
Cognitive mental status and communication	1 = At least one impairment	1/8	76.45
Cognition	1 = Low cognition	1/48	40.47
Memory	1 = Poor	1/48	40.37
Vision	1 = Bad	1/48	40.25
Hearing	1 = Poor	1/48	20.14
Sadness	1 = Poor	1/48	0.32
Depression	1 = Yes	1/48	34.48
Medical condition	1 = At least one impairment	1/8	43.44
Chronic disease	1 = Yes	1/16	24.83
Physical disabilities	1 = Yes	1/16	22.98
Sources of care	1 = At least one impairment	1/8	39.75
Access to caregiver	1 = No	1/16	29.57
Living arrangement	1 = Live alone and far away from children	1/16	15.84
Home settings	1 = At least one impairment	1/8	28.92
Elevator in a four-story apartment	1 = No	1/16	3.92
Accessibility	1 = No	1/16	25.08
Social interactions	1 = At least three impairments	1/8	54.44
Interacted with friends	1 = No	1/40	65.92
Community club	1 = No	1/40	83.98
Provide help to friends	1 = No	1/40	95.58
Outdoor activities	1 = No	1/40	94.08
Community-related organization	1 = No	1/40	98.65

*1, disability or impairment in this index; 0, without any impairment in this index*.

**Table 8:** The contribution of multidimensional disability indexes (M_0_) after adjustment.

		**The contribution rate (%) of M**_**0**_							
**Cut-off**	**M_**0**_**	**Self-care**	**Cognition and communication**	**Ability of processing diseases**	**Motor ability**	**Medical condition**	**Sources of care**	**Home settings**	**Social**
*k* = 1	0.5654	0.0552	0.1837	0.1571	0.1255	0.1044	0.0955	0.0693	0.2092
*k* = 2	0.5499	0.0556	0.1841	0.1571	0.1260	0.1049	0.0956	0.0695	0.2073
*k* = 3	0.5454	0.0580	0.1836	0.1565	0.1289	0.1066	0.0961	0.0697	0.2006
*k* = 4	0.4659	0.0656	0.1794	0.1535	0.1350	0.1097	0.0983	0.0709	0.1876
*k* = 5	0.3757	0.0833	0.1692	0.1477	0.1426	0.1128	0.1007	0.0727	0.1710
*k* = 6	0.2123	0.1051	0.1555	0.1403	0.1434	0.1195	0.1065	0.0764	0.1534
*k* = 7	0.0752	0.1215	0.1394	0.1327	0.1352	0.1281	0.1144	0.0897	0.1390
*k* = 8	0.0129	0.125	0.125	0.125	0.125	0.125	0.125	0.125	0.125

(2) Bourguignon and Chakravarty index (BC^a^)

Table 9 presented that the BC^a^ index value of multidimensional disability of people by different genders, ages, and places of residence ranges from 0.277 to 0.503. Older people who were female, living in rural areas, and older than 65 years have higher BC^a^ index. This showed a consistency with the findings of AF methods. Overall, older people have 2 ~ 6 dimensions of disability; when *k* = 1, 5, and 6, the proportion of multi-dimensional disability in older adults reached 0.566, 0.376 and 0.212, respectively, indicating that multidimensional disability rate by AF is reliable and accurate. Thus, we considered BC^a^ as part of the results for sensitivity analysis to further support our conclusions.

**Table 9:** BC^a^ indexes among older people by different age, gender, and place of residence.

**Group**	**Estimate**	**STE**	**LB**	**UB**
**Age**				
Over 65	0.355	0.008	0.342	0.368
Less 65	0.277	0.010	0.260	0.293
**Sex**				
Male	0.305	0.009	0.290	0.319
Female	0.349	0.009	0.334	0.364
**Place of residence**				
Rural	0.289	0.006	0.278	0.299
Urban	0.503	0.018	0.472	0.533
**Total**	0.327	0.006	0.316	0.337

*Estimate: mean value; STE: the standard deviation; LB: 25% CI BC^a^; UB: 75% CI BC^a^*.

#### An Empirical Model for Influencing Factors of Multidimensional Disability

The results of the Breusch-Pagan test in Table 10 presented that χ^2^ = 503.734, *P* < 0.001. It indicates that there is autocorrelation in the eight dimensions of the interference terms ε, which is suitable for the analysis by Seemingly Unrelated Regression. Table 11 showed that there were differences in the demographic characteristics and biomarkers of multidimensional disability among older adults in terms of gender, age, resident place, BMI, educational level, formal care, and creatinine. Those older people who are female, aged over 65, and divorced have significant advantages in the dimensions of sources of care and home settings. The higher their average BMI was, the worse movement ability and home setting for older people would be. Those older people who are living in urban areas have a higher degree of deprivation in the dimension of care resources.

**Table 10:** Residual matrix analysis of each dimension.

	**Self-care**	**Cognition and communication**	**Ability of processing diseases**	**Movement**	**Medical condition**	**Sources of care**	**Home settings**	**Social interactions**
Self-care	1.000							
Cognition and communication	0.123	1.000						
Ability of processing diseases	−0.013	0.012	1.000					
Movement	0.197	0.183	−0.037	1.000				
Medical condition	−0.007	0.038	0.091	−0.020	1.000			
Sources of care	0.045	0.045	0.019	0.041	−0.004	1.000		
Home settings	−0.022	0.030	−0.012	−0.011	0.033	−0.041	1.000	
Social interactions	0.029	0.039	0.014	0.028	0.012	−0.007	−0.0129	1.000

*Breusch-Pagan test: χ^2^: 503.734, P < 0.001*.

**Table 11:** Analysis of influencing factors of multidimensional disability.

	**Self-care**	**Cognition and communication**	**Ability of processing diseases**	**Movement**	**Medical condition**	**Sources of care**	**Home settings**	**Social interactions**	**Total (OLS)**
Age	0.0008*** (<0.001)	0.0005*** (<0.001)	0.0004** (0.006)	0.0011*** (<0.001)	0.0006*** (<0.001)	−0.0001 (0.381)	−0.0005*** (<0.001)	0.0002 (0.068)	0.0029*** (<0.001)
Female	0.0055*** (<0.001)	0.0146*** (<0.001)	0.0019 (0.352)	0.0188*** (<0.001)	−0.0106*** (<0.001)	−0.0027 (0.177)	−0.0014*** (0.472)	0.0021 (0.146)	0.0282*** (<0.001)
BMI	−0.0005* (0.022)	−0.0004** (0.007)	−0.0009*** (<0.001)	0.0007*** (<0.001)	−0.0013*** (<0.001)	0.0001 (0.818)	0.0004*** (0.076)	−0.0005*** (0.001)	−0.0021** (0.001)
Urban	−0.0115*** (<0.001)	−0.0132*** (<0.001)	−0.0044 (0.059)	−0.0022 (0.305)	−0.0063** (0.008)	0.0068** (0.004)	0.0021 (0.357)	−0.0069*** (<0.001)	−0.0288*** (<0.001)
**Marital status**									
Divorced	0.0005 (0.786)	0.0032 (0.118)	−0.0001 (0.968)	0.0011 (0.605)	0.0024 (0.335)	−0.0094*** (<0.001)	−0.0027 (0.256)	−0.0026 (0.143)	−0.0077 (0.256)
Single	−0.0006 (0.931)	0.0091 (0.266)	0.0051 (0.603)	−0.0047 (0.593)	0.0227* (0.023)	0.0319*** (0.001)	−0.0094 (0.329)	−0.0063 (0.377)	0.0477 (0.081)
**Educational level**									
Primary school	−0.0027 (0.089)	−0.0163*** (<0.001)	−0.0055* (0.013)	−0.0026 (0.182)	−0.008*** (<0.001)	0.0029 (0.192)	−0.003 (0.164)	−0.0047** (0.003)	−0.0398*** (<0.001)
High school	−0.0097*** (<0.001)	−0.0344*** (<0.001)	−0.007** (0.017)	−0.0119*** (<0.001)	−0.014*** (<0.001)	0.003 (0.320)	−0.0001 (0.963)	−0.0116*** (<0.001)	−0.0857*** (<0.001)
Bachelor and above	−0.0047 (0.345)	−0.0354*** (<0.001)	−0.0067 (0.327)	−0.025*** (<0.001)	−0.0179** (0.010)	0.021** (0.002)	0.0209** (0.002)	−0.0125* (0.012)	−0.0603** (0.001)
per-capital annual household income	−0.0000 (0.427)	−0.0000 (0.119)	0.0000* (0.014)	0.0000 (0.680)	−0.0000 (0.933)	0.0000 (0.083)	0.0000 (0.994)	0.0000 (0.060)	0.0000 (0.255)
Formal care	0.0002*** (<0.001)	0.0001*** (<0.001)	0.0000 (0.173)	0.0001*** (<0.001)	0.0000 (0.119)	0.0000** (0.001)	0.0000 (0.494)	0.0000** (0.006)	0.0004*** (<0.001)
Informal care	0.0000 (0.558)	−0.0000 (0.429)	−0.0000 (0.072)	−0.0000 (0.485)	0.0000 (0.215)	−0.0001** (0.008)	0.0001* (0.012)	−0.0000 (0.601)	−0.0000 (0.532)
systolic pressure	−0.0001 (0.137)	0.0000 (0.683)	−0.0001 (0.149)	0.0000 (0.748)	0.0000 (0.334)	0.0000 (0.508)	−0.0001 (0.26)	0.0000 (0.477)	−0.0002 (0.143)
diastolic pressure	0.0003** (0.001)	0.0000 (0.620)	0.0001 (0.201)	0.0001 (0.269)	0.0001* (0.401)	−0.0003* (0.012)	0.0002 (0.057)	0.0001 (0.265)	0.0006* (0.035)
Creatinine	0.0074*** (<0.001)	−0.0007 (0.773)	−0.0015 (0.623)	0.0002 (0.926)	0.0075* (0.013)	0.0071* (0.018)	−0.0007 (0.817)	0.0003 (0.881)	0.0197* (0.017)
Total cholesterol	0.0000 (0.921)	0.0000 (0.781)	0.0000* (0.026)	0.0000 (0.287)	0.0000 (0.428)	0.0000 (0.706)	0.0000 (0.971)	0.0000 (0.438)	0.0001 (0.070)
C-reactive protein (hsCRP)	0.0000 (0.786)	0.0001 (0.284)	−0.0001 (0.212)	0.0000 (0.944)	0.0001 (0.575)	0.0000 (0.742)	0.0001 (0.279)	0.0001 (0.304)	0.0002 (0.512)
Glycated hemoglobin (HbA1c)	0.0010 (0.149)	0.0000 (0.987)	−0.0011 (0.221)	−0.0001 (0.934)	−0.0016 (0.088)	−0.0015 (0.104)	−0.001 (0.258)	−0.0002 (0.744)	−0.0046 (0.074)
Hemoglobin	−0.0001 (0.576)	−0.0004 (0.148)	0.0003 (0.284)	0.0002 (0.439)	0.0007* (0.026)	0.0003 (0.301)	0.0001 (0.652)	0.0003 (0.217)	0.0014 (0.079)

***P < 0.001; **P < 0.01; *P < 0.05; BMI: Body Mass Index; HbA1c: Glycated hemoglobin; hsCRP: high sensitive C-reactive protein; the reference of aged over 65, male, rural, married, and illiteracy.

## Discussion

The present study was the first to construct MDI linked with individual, sources, and society capabilities in China, based on the A-F method. To verify the sensitivity of the threshold of index and the dimension, the overall trend and the results of decomposition of the multi-dimensional disability index were found to be basically consistent with the previous analysis after altering the threshold of index and the dimensions, indicating that the constructed index system was robust with high sensitivity and reliability.

Persons with impairment in motor ability, cognitive mental status and communication skills, ability of processing disease, and social interactions were significantly less likely to access any opportunity to achieve good health and escape morbidity. A relatively lenient threshold of the dimension of self-care was defined as disability if there existed at least one functional impairment in the dimension. The results showed that the measurement results of daily living ability disability were similar to another study (53). However, the higher impairment of motor ability, cognitive mental status and communication skills, ability of processing disease, and social interactions had larger contribution rates to multidimensional disability than self-care ability. As for motor ability, continuous stair climbing measured by mobility contributed the most to the limitation of motor ability, and older adults with limited stair climbing function accounted for 50.83%, followed by balance (35.67%). It indicated that stair climbing dysfunction has a great impact on the life of older adults. Another study also estimated that subjects who often climb stairs have a lower risk of coronary heart disease than those who do not (54). However, climbing stairs requires more muscle strength and energy than walking on flat ground, giving the body four Metabolic equivalent (METs) of moderately intense exercise, thus, stair climbing is not suitable for rehabilitation and endurance exercise for older adults (55). In addition, if there was no elevator in their living space, older adults who have insufficient physical endurance to climb stairs would give up outdoor activities, which results in further functional impairment.

As another kind of motor ability, balance dysfunction in older adults is regulated by the sensory system, nervous system, skeletal muscle system, spirit, and psychology. From the external aspect, balance function impairment in older adults results in increasing the incidence of falls, high hospitalization rate, and high mortality, which brings serious burden to individuals and society (56). From the internal body structure analysis, balance mainly depends on the central nervous system from proprioception vestibular sense and the sense of vision system information integration and regulation to maintain the balance of internal body (57). Once problems happen to a certain sense organ, the other two sensory organs will accordingly deploy compensatory strategies to keep the balance of the body, otherwise, a sensory system or nervous system will be damaged and degenerate. Therefore, attention should be paid to the imbalance of the limbs of older adults, the causes should be found in time, and treatment measures should be taken. At the same time, the government should popularize sports therapy, such as taijiquan (58), gymnastics (59), and balance dance (60), which are important for preventing balance dysfunction in older adults and maintaining the normal functions of all senses.

In the process of dealing with the demands and pressures caused by diseases, the self-treatment method of older adults is mainly to buy over-the-counter western medicine and traditional Chinese herbal medicine or to use traditional methods for treatment. This study found that the self-treatment rate of older adults within 1 month was 51.4% in 2011, lower than the self-treatment rate of all older adults in 2015 (61). With the aggravation of aging, China's disease spectrum is shifting to geriatric diseases and chronic diseases, because of the long course of chronic diseases, which need continuous treatment and long-term drug control. In addition, the popularization of compulsory education has reduced the number of illiterate people and increased the awareness rate of medical care knowledge of older adults and some common diseases. Therefore, the main reason for the increase in the utilization rate of self-treatment services might be older adults' self-perception that their conditions are not serious and the characteristics of chronic diseases. However, self-treatment rates having risen only 2% in 4 years suggests that the government and society should advocate to improve older adults health care system as soon as possible, improve older adults health literacy, and help old people actively deal with long-term health problems.

In addition, as people with certain social attributes get to have insufficient external resources (like assistive device and infrastructure, etc.), they suffered restricted autonomy and social participation that will further deteriorate the cognitive ability, daily living ability, and disease status of older adults (62). The poor home settings and lack of care sources resulted in increased probability of undergoing poorer treatment, and certainly the risk of a tumor or comorbidities. The results of this study show that the contribution rate of living environment to multidimensional disability is high. Every year, 55.2% of older adults who are injured due to falls occur indoors, and 30.3% of them suffer from fractures, most of which require surgical treatment (63). The reason behind this is the lack of ground anti-slip and anti-trip handrails in bathrooms, toilets, and barrier-free living environment and other facilities. As mentioned in the introduction of *residential architectural design for older adults*, the ideal living environment for older adults is in a basic living space, according to the comfort scale of older adults, to meet the requirements of health and application, and on this basis, to further improve the safety, comfort, and health, and to select materials, equipment, and products with good performance and quality (64).

The more access a family has to caregiver resources, the better the healthy development of older adults is. The current rate of ADL dysfunction in older adults is about 10~20% (65, 66), but the rate of ADL disability in this study was 23.72%, which was less than the disability caused by poor accessibility of caregivers (29.57%) and lack of accessibility (25.08%). It turned out that the older people with only ADL impairment belong to a minority group. Even more significant is the fact those who are unattended, with poor accessibility, should urgently have their care needs addressed. It has been proven that those unattended who live alone without caregivers are less likely to get proper treatment, and certainly increases the risk of a tumor or returning illness (67).

It was suggested that the government should encourage the development of multiple service resources to meet the diverse needs of the disabled older population, which included improving their motor capacity by providing assistive devices to enhance their activity capacity, timely provision of services targeting the cognitive function and communication ability of older adults, and optimization of the living conditions to enable the caregivers to release stress by the advanced evaluating of home care resources for those care recipients who are disadvantaged in the dimension of life. The experience of Germany can be used for reference to develop diverse community care resources and build a home-based community environment (68, 69), and improve the feasible ability set of disabled older adults through “aging in place” and “nearby assistance,” so as to meet the diversified pension needs of older adults and provide more dignified long-term care services for disabled older adults.

The overall multidimensional disability status indicates that older people who are female, aged over 65, with lower BMI, living in rural areas, with a lower education level, getting more formal care, and with a relatively higher creatinine, face a higher risk of deprivation in overall multidimensional disability and poor quality of life. These findings confirmed that all walks of life should pay attention to the multidimensional disability of older adults who are living in rural areas, with a lower level of education, since they are in a severe deprivation in the dimension of social interaction. The existence of a good social network of acquaintances results in better care and health management, which can alleviate the multidimensional disability level of older adults (70). The results also presented a positive correlation between creatinine and deprivation of medical condition of multidimensional disability. Creatinine is a toxin produced by muscle metabolism. In human muscles, creatinine is slowly formed mainly through irreversible non-enzymatic dehydration reactions, which is then released into the bloodstream and cleared by the kidneys. When kidney function is impaired, the glomerular filtration rate decreases and blood creatinine values are high. Increased creatinine levels lead to symptoms such as fatigue, weakness, back pain, and loss of appetite (71). This concludes that potential biomarker factors could be a clinical predictor for earlier designing personalized intervention to delay the progression of disability and prevent its occurrence.

Although we are confident that age is a strong predictor of impairment, we did not find any significant association between cognitive mental status and communication skills and social interaction with age; thus it was illustrated that the two dimensions were not affected by age, which is the opposite of previous research results (72). The reason behind is that this study focuses on a range of ages (80). Additionally, based on China's traditional filial piety culture, the older population have the responsibility to take care of their grandchildren. As those older people achieve certain social and economic status with their family relationships becoming more stable with age, they will get less stress to care for grandchildren and an urgent need to communicate and engage in social and cultural activities.

The MDI makes it possible to accurately measure the gap between rural and urban populations in terms of multidimensional disability situation. Zhang et al. (73) argued that the degree, scale, and care cost of disability of older adults in urban areas were higher than those in rural areas by using single-dimension ADL disability. However, the results of multidimensional disability measured in this study showed that older adults living in rural areas were worse in all dimensions of disabilities and have a higher disability rate than those in urban areas due to the lack of home environment and medical infrastructure. They also have not received timely and effective long-term care and pension financial subsidies, subsequently resulting in a vicious circle. In light of the above findings, it can be argued that multi-dimensional deprivation of older adults should be considered comprehensively, based on personal resources and social aspects from a broader perspective to calculate the disability scale and care cost of older adults in China. At the same time, the government and relevant departments should pay more attention to the evaluation of home care resources for older adults in rural areas, help older adults who are disadvantaged in the dimension of life to improve their family living conditions, and mitigate and limit the extension of disability in terms of resources and environment.

The analysis showed in the factors of social interaction, cognitive mental status and communication skills, motor ability, and the ability to process diseases accounted for a higher proportion in terms of multidimensional disability in older adults. The assessment of the disability degree of older adults from the perspective of multidimensional disability will contribute to broader measures to improve their social participation and mental health, which will enhance the health literacy and their ability to use assistive devices to deal with diseases, and further strengthen home environment improvement guidance. Especially for older adults with multiple cognitive and psychological impairments, it is necessary to provide relevant policy support to overcome the health deterioration caused by da isadvantaged environment and impaired social participation of older adults, so as to improve the quality of life and dignity in later life.

## Limitations

Nevertheless, there existed two limitations to this study that need to be addressed: the first important one is that the multidimensional disability indexes need external validation in other countries and settings, although the Capability approach and geriatric medicine knowledge and were thought to be a well-based proof of concept. Secondly, the information of the binary ordered measurement of individual deprivation in different disability dimensions is limited. However, by fully mining and measuring the indicators information, we assume that each basic attribute has priority over the non-basic attribute categories, and the importance of each dimension in each category also varies. In addition, individuals with the same level of disability in some subjects but with different degrees of insufficiency in other aspects should be different. Based on the adjusted multidimensional deprivation rules by Dhongde, Pattanaik, and Bourguignon and Chakravarty, we use DASP to BC^a^ indexes to further solve the above problems. Finally, the construct itself consists of three aspects and eight dimensions with 29 indexes, which are debatable. However, we have adopted the OLS model to analyze the factors affecting overall dimensional disability to further explore more information and characteristics among dimensions and make a confirmation of this construct.

## Conclusions

Our data study suggested that the combined multiple variables central to the development of disability is superior to measurement of only one or two aspects, ADL or IADL. Through constructing a multi-dimensional disability evaluation index system, the study provides a first step toward a more precise and comprehensive evaluation for older adults in a home-based community. Our study also paves the way for heterogeneity analysis of older people aiming to predict and analyze the care needs of older people and provide precision nursing in the future.

## Data Availability Statement

The original contributions presented in the study are included in the article/Supplementary Material, further inquiries can be directed to the corresponding author.

## Author Contributions

YH, LZ, and YF worked together and provide the conception and conduct study design of this work. YH analyzed and interpreted the data and drafted the manuscript. YF and LZ supervised and revised the manuscript. All authors have revised, read, and approved the final manuscript and agree to be accountable for all aspects of the work in ensuring that questions related to the accuracy or integrity of any part of the work are appropriately investigated and resolved.

## Conflict of Interest

The authors declare that the research was conducted in the absence of any commercial or financial relationships that could be construed as a potential conflict of interest.

## Publisher's Note

All claims expressed in this article are solely those of the authors and do not necessarily represent those of their affiliated organizations, or those of the publisher, the editors and the reviewers. Any product that may be evaluated in this article, or claim that may be made by its manufacturer, is not guaranteed or endorsed by the publisher.

## References

[1] Taş U Steyerberg EW Bierma-Zeinstra SM Hofman A Koes BW Verhagen APJBG. Age, gender and disability predict future disability in older people: the rotterdam study. BMC Geriat. (2011) 11:22. 10.1186/1471-2318-11-22 21569279 PMC3224098

[2] Venturelli M Reggiani C Richardson RS Schena F. Skeletal muscle function in the oldest-old: the role of intrinsic and extrinsic factors. Exerc Sport Sci Rev. (2018) 46:188–94. 10.1249/JES.0000000000000155 29672349 PMC6005743

[3] Newman AB Sanders JL Kizer JR Boudreau RM Odden MC Zeki Al Hazzouri A. Trajectories of function and biomarkers with age: the CHS all stars study. Int J Epidemiol. (2016) 45:1135–45. 10.1093/ije/dyw092 27272182 PMC5841627

[4] Trani JF Bakhshi P Brown D Lopez D Gall F. Disability as deprivation of capabilities: estimation using a large-scale survey in morocco and tunisia and an instrumental variable approach. Soc Sci Med. (2018). 211:48–60. 10.1016/j.socscimed.2018.05.033 29890357

[5] Ortman JM VV Hogan H. An Aging Nation: The Older Population in the United States. Washington, DC: United States Census Bureau, Economics and Statistics Administration, US Department of Commerce (2014).

[6] Zhang W Marcus WF Du P. Decline and cohort differences in activities of daily living of the Chinese oldest-old. Populat Res. (2019) 43:3–16. Available online at: http://d.oldg.wanfangdata.com.cn/Periodical_rkyj201903001.aspx

[7] Zhoumei. Study on the Theoretical Model of Self-Care Defect for Older Adults in Home Care in Shanghai. (Doctoral dissertation), Second Military Medical University, Shanghai, China. (2012). 10.7666/d.y2111084

[8] Anonymous. The results of the fourth Sample survey on the living conditions of the Elderly in Urban and rural China are released. Chin J Gerontol. (2016) 21:60–8. Available online at: https://wenku.baidu.com/view/f6fa2833fc00bed5b9f3f90f76c66137ee064f1b.html

[9] Chatterji S Byles J Cutler D Seeman T Verdes E. Health, functioning, and disability in older adults–present status and future implications. Lancet. (2015) 385:563–75. 10.1016/S0140-6736(14)61462-8 25468158 PMC4882096

[10] AbizandaPRomeroLSánchez-JuradoPMMartínez-ReigMAlfonso-SilgueroSARodríguez-MañasL. Age, frailty, disability, institutionalization, multimorbidity or comorbidity. Which are the main targets in older adults? J Nutr Health Aging. (2014) 18:622–7. 10.1007/s12603-014-0033-324950154

[11] Bonaga B Sánchez-Jurado PM Martínez-Reig M Ariza G Rodríguez-Mañas L Gnjidic D. Frailty, polypharmacy, and health outcomes in older adults: the frailty and dependence in albacete study. J Am Med Directors Assoc. (2018) 19:46–52. 10.1016/j.jamda.2017.07.008 28899661

[12] Tamayo M Besoaín Á Rebolledo J. [Social determinants of health and disability: updating the model for determination]. Gaceta sanitaria. (2018) 32:96–100. 10.1016/j.gaceta.2016.12.004 28274622

[13] Pfeiffer D. The conceptualization of disability.:In Altman BM, Barnartt S, editors. Exploring Theories Andexpanding Methodologies: Research in Social Science and Disability. Oxford: Elsevier (2011). p. 225–9.

[14] Dubois JL Trani JF. Enlarging the capability paradigm to address the complexity of disability. ALTER Eur J Disabil Res. (2009) 3:2–8. 10.1016/j.alter.2009.04.003

[15] Buchanan A. Genetic intervention and the morality of inclusion. In: Buchanan A, Brock DW, Daniels N, Wikler D, editors. From Chance to Choice: Genetics and Justice. Cambridge: Cambridge University Press (2000). p. 258–303.

[16] Crasto CL Semba RD Sun K Cappola AR Bandinelli S Ferrucci L. Relationship of low-circulating “anti-aging” klotho hormone with disability in activities of daily living among older community-dwelling adults. Rejuvenat Res. (2012) 15:295–301. 10.1089/rej.2011.1268 22530731 PMC3388499

[17] Organization WH. ICF-The International Classification of Functioning, Dlisability and Health. Geneva: World Health Organization (2005).

[18] Andersen K Nybo H Gaist D Petersen HC McGue M Jeune B. Cognitive impairment and mortality among nonagenar-ians: the Danish 1905 cohort survey. Dement Geriatr Cogn Disord. (2002) 131:56–63. 10.1159/000048647 11893837

[19] Franceschi C Motta L Valensin S Rapisarda R Franzone A Berardelli M. Do men and women follow different trajectories to reach extreme longevity? Aging Clin Exp Res. (2000) 12:77–84. 10.1007/BF03339894 10902049

[20] Gondo Y Hirose N Arai Y Inagaki H Masui Y Yamamura K. Functional status of centenarians in tokyo, japan: developing better phenotypes of exceptional longevity. J Gerontol A. (2006) 61:305–10. 10.1093/gerona/61.3.305 16567382

[21] Andersen-Ranberg K Schroll M Jeune B. Healthy centenarians do not exist, but autonomous centenarians do: a population-based study of morbidity among Danish centenarians. J Am Geriatr Soc. (2001) 49:900–8. 10.1046/j.1532-5415.2001.49180.x 11527481

[22] Evert J Lawler E Bogan H Perls T. Morbidity profiles of centenarians: survivors, delayers, and escapers. J Gerontol A Biol Sci Med Sci. (2003) 58:232–7. 10.1093/gerona/58.3.M232 12634289

[23] Hoogendijk EO Romero L Sánchez-Jurado PM Flores Ruano T Viña J Rodríguez-Mañas L. A new functional classification based on frailty and disability stratifies the risk for mortality among older adults: the FRADEA Study. J Am Med Dir Assoc. (2019) 20:1105–10. 10.1016/j.jamda.2019.01.129 30853426

[24] Trani JF Browne J Kett M Bah O Morlai T Bailey N. Access to health care, reproductive health and disability: a large scale survey in Sierra Leone. Soc Sci Med. (2011). 73:1477–89. 10.1016/j.socscimed.2011.08.040 22014873

[25] Mitra S. Disability, Health and Human Development. New York, NY: Palgrave Macmillan (2017).

[26] Sen A. Conceptualizing and Measuring Poverty. In: David G, Ravi K, editors. Poverty and Inequality. Stanford, CA: Stanford University Press (2006).

[27] Burchardt T. Capabilities and disability: the capabilities framework and the social model of disability. Disability Soc. (2004) 19:735–51. 10.1080/0968759042000284213

[28] Mitra S. The capability approach and disability. J Disability Policy Stud. (2006) 16:236–47. 10.1177/10442073060160040501

[29] Cosco TD Stephan BC Brayne C. (Unsuccessful) binary modeling of successful aging in the oldest-old adults: a call for continuum-based measures. J Am Geriatr Soc. (2014) 62:1597–8. 10.1111/jgs.12958 25116987

[30] Jean-Francois T Parul B Sarah MT Dominique L Fiona G. Disability and poverty in morocco and tunisia: a multidimensional approach. J Hum Dev Capabil. (2015) 16:518–48. 10.1080/19452829.2015.109180829890357

[31] Luzzi GF Flückiger Y Weber S. A cluster analysis of multidimensional poverty in Switzerland. In: Kakwani N, Silber J, editors. Quantitative Approaches to Multidimensional Poverty Measurement. London: Palgrave Macmillan UK (2008). p. 63–79.

[32] Bourguignon F Chakravarty SR. The measurement of multidimensional poverty. J Econ Inequal. (2003) 1:25–49. 10.1023/A:1023913831342

[33] Lugo MA Maasoumi E. Maasoumi.Multidimensional Poverty Measures from an Information Theory Perspective[M]. Oxford Poverty & Human Development Initiative Working Paper (2009).

[34] Tsui KY. Multidimensional poverty indices. Soc Choice Welfare. (2002) 19:69–93. 10.1007/s355-002-8326-3

[35] Alkire S Foster J. Understandings and misunderstandings of multidimensional poverty measurement. J Econ Inequal. (2011) 9:289–314. 10.1007/s10888-011-9181-4

[36] Alkire S Foster J. Counting and Multidimensional Poverty Measurement. OPHI Working Paper 7, Oxford: Oxford University (2007).

[37] Alkire S Foster J. Counting and multidimensional poverty measurement. J Public Econ. (2011) 95:476–87. 10.1016/j.jpubeco.2010.11.006

[38] Sabina Alkire JF. Dimensional and Distributional Contributions to Multidimensional Poverty. OPHI Working Papers 100, Queen Elizabeth House, University of Oxford (2016).

[39] Alkire S Shen Y. Exploring Multidimensional Poverty in China. Oxford: Oxford Poverty & Human Development Initiative: University of Oxford (2016).

[40] Dhongde S Haveman R. Multi-dimensional deprivation in the U.S. Soc Indicat Res. (2017) 133:477–500. 10.1007/s11205-016-1379-1

[41] Dhongde S Li Y Pattanaik PK Xu Y. Binary data, hierarchy of attributes, and multidimensional deprivation. J Econ Inequal. (2016) 14:363–78. 10.1007/s10888-016-9336-4

[42] Filmer D Scott K. Assessing asset indices. Demography. (2012) 49:359–92. 10.1007/s13524-011-0077-5 22135117

[43] Bossert W Chakravarty SR D'Ambrosio C. Multidimensional poverty and material deprivation with discrete data. In: Chakravarty SR, editor. Poverty, Social Exclusion and Stochastic Dominance. Singapore: Springer Singapore (2019). p. 191–209.

[44] Satya Chakravarty CDA. The measurement of social exclusion, international association for research in income and wealth. Rev Income Wealth. (2006) 52:377–98. 10.1111/j.1475-4991.2006.00195.x

[45] Aaberge R Andrea B. Multidimensional Poverty Inequality. Bank of Italy Temi di Discussione (Working Paper) No. 976. (2014). Available online at: https://ssrn.com/abstract=2550775 (accessed January 17, 2015).

[46] Bosmans K Lauwers L Ooghe E. Prioritarian poverty comparisons with cardinal and ordinal attributes. Scand J Econ. (2018) 120:925–42. 10.1111/sjoe.12238

[47] Duclos JY Luca T. Multidimensional Poverty Indices: A Critical Assessment. PEP Working Paper Series. (2016). Available online at: https://ssrn.com/abstract=2718374 (accessed July 16, 2018).

[48] Sánchez-García JF Sánchez-Antón MdC Badillo-Amador R Marco-Gil MdC Llinares-Ciscar JV Álvarez-Díez S. A new extension of bourguignon and chakravarty index to measure educational poverty and its application to the OECD Countries. Soc Indicat Res. (2019) 145:479–501. 10.1007/s11205-019-02115-x

[49] Zhang C Lei X Strauss J Zhao Y. Health insurance and health care among the mid-aged and older Chinese: evidence from the National Baseline Survey of CHARLS. Health Econ. (2017) 26:431–49. 10.1002/hec.3322 26856894 PMC4980285

[50] Zhao Y Hu Y Smith JP Strauss J Yang G. Cohort profile: the China health and retirement longitudinal study (CHARLS). Int J Epidemiol. (2014) 43:61–8. 10.1093/ije/dys203 23243115 PMC3937970

[51] Eaton WW Smith C Ybarra M Muntaner C Tien A. Center for epidemiologic studies depression scale: review and revision (CESD and CESD-R). In: Maruish ME, editor, The Use of Psychological Testing for Treatment Planning and Outcomes Assessment: Instruments for Adults. Lawrence Erlbaum Associates Publishers (2004). p. 363–77.

[52] Alkire S Santos ME. Acute Multidimensional Poverty: A New Index for Developing Countries. Oxford: Oxford Poverty & Human Development Initiative (OPHI) Working Paper, United Nations Development Programme Human Development Report Office Background Paper (2010).

[53] Yang MX Lu B Mi H. Research on the disability prevalence trend and the influencing factors for china's elderlies———based on the 2000,2006 and 2010. SSAPUR DATA. Popul Dev. (2018) 24:97–106.

[54] Morris JN Heady J Raffle P Roberts C Parks JJTl. Coronary heart-disease and physical activity of work. Lancet. (1953) 262:1111–20. 10.1016/S0140-6736(53)91495-0 13110075

[55] Ainsworth BE HW Herrmann SD Meckes N Bassett DR Jr Tudor-Locke C. Compendium of physical activities: a second update of codes and MET values. Med Sci Sports Exerc. (2011) 2012:1575–81. 10.1249/MSS.0b013e31821ece12 21681120

[56] Auvinet B Touzard C Montestruc F Delafond A Goeb V. Gait disorders in the elderly and dual task gait analysis: a new approach for identifying motor phenotypes. J Neuroeng Rehabil. (2017) 14:7. 10.1186/s12984-017-0218-1 28143497 PMC5282774

[57] Sibley KM Mochizuki G Lakhani B McIlroy WE. Autonomic contributions in postural control: a review of the evidence. Rev Neurosci. (2014) 25:687–97. 10.1515/revneuro-2014-0011 24854534

[58] Wei G. Effect of Tai Chi Exercise on Prefrontal Executive Function in Older Adults: A Multimodal Magnetic Resonance Study. Beijing: Institute of Psychology, Chinese Academy of Sciences.

[59] Jun D. Effect of body-building gymnastics based on core strength training on balance ability of the elderly. Chin J Sports Med. (2017) 36:992–4.

[60] Jiajiao J. Study on the Effect of Aerobic Dance Exercise on Balance Ability of Middle-Aged and Elderly. Chengdu: Chengdu University of Physical Education. (2021).

[61] Haiyan Y. Research on Medical Expenses of the Elderly and Its Influencing Factors. Guizhou: Guizhou University of Finance and Economics (2019).

[62] Peng D Juanjuan S Wenjuan Z Xuehui W. The demands of old-age care and the family and social resources for the Chinese elderly: a study based on 2014. China longitudinal aging social survey. Populat Res. (2016) 40:49–61. Available online at: http://d.g.wanfangdata.com.cn/Periodical_rkyj201606004.aspx

[63] Liu Hua LB. Research on the key factors influencing the demand of urban residential renovation for the aged. Reform Strategy. (2012) 28:175–8. 10.16331/j.cnki.issn1002-736x.2012.03.01631914574

[64] Xiufeng W. Thinking about the renovation of existing residential buildings for old age. Anhui Arch. (2014) 21:12–3. 10.3969/j.issn.1007-7359.2014.03.003

[65] Zhang WM. Disability level of the chinese elderly:comparison from multiple data sources. Populat Res. (2015) 39:34–47.

[66] Jun T. How many disabled elderly people there are in China? China Soc Security. (2016) 6:38–40.

[67] Huang RY Lin YH Lin SY Li YN Chiang CS Chang CW. Magnetic ternary nanohybrids for nonviral gene delivery of stem cells and applications on cancer therapy. Theranostics. (2019) 9:2411–23. 10.7150/thno.29326 31149052 PMC6531296

[68] Ache P Fedrowitz M. The development of co-housing initiatives in germany. Built Environment. (2012) 38:395. 10.2148/benv.38.3.395

[69] Jansen KSK Bölting T. Gemeinsam Statt Einsam! GemeinschaftlicheWohnprojekte für Ältere[EB/OL]. (2009). Available online at: http://www.aqnrw.de/media/kbw_gemeinsam_statt_einsam.pdf (accessed January 1, 2022).

[70] Nakamura H Managi S. Effects of subjective and objective city evaluation on life satisfaction in Japan. J Cleaner Product. (2020) 256:120523. 10.1016/j.jclepro.2020.12052330975019

[71] Núñez J Garcia S Núñez E Bonanad C Bodí V Miñana G. Early serum creatinine changes and outcomes in patients admitted for acute heart failure: the cardio-renal syndrome revisited. Eur Heart J Acute Cardiovasc Care. (2017) 6:430–40. 10.1177/2048872614540094 25080512

[72] Yuling Q Xiumin Z Xiuxin S Yuting H Hang G Wei L. Social support status and influencing factors of the elderly in urban community. Chin General Pract. (2016) 19:3099–102.21323182

[73] Zhang L Fu S Ya F. Prediction of disability scale and care cost of the elderly in urban and rural China from 2020 to 2050. Chin J Health Stat. (2021) 38:39–42. 10.3390/su12072598

