# Supplementary material for: Multidimensional Disability Evaluation and Confirmatory Analysis of Older Adults in a Home-Based Community in China
Source: Front Public Health. 2022 Jun 21;10:899303. doi: 10.3389/fpubh.2022.899303 (PMC9253425; doi:10.3389/fpubh.2022.899303)
Supplement: Supplementary file 1 [file Presentation_1.pdf]

Supplementary Figures and Tables

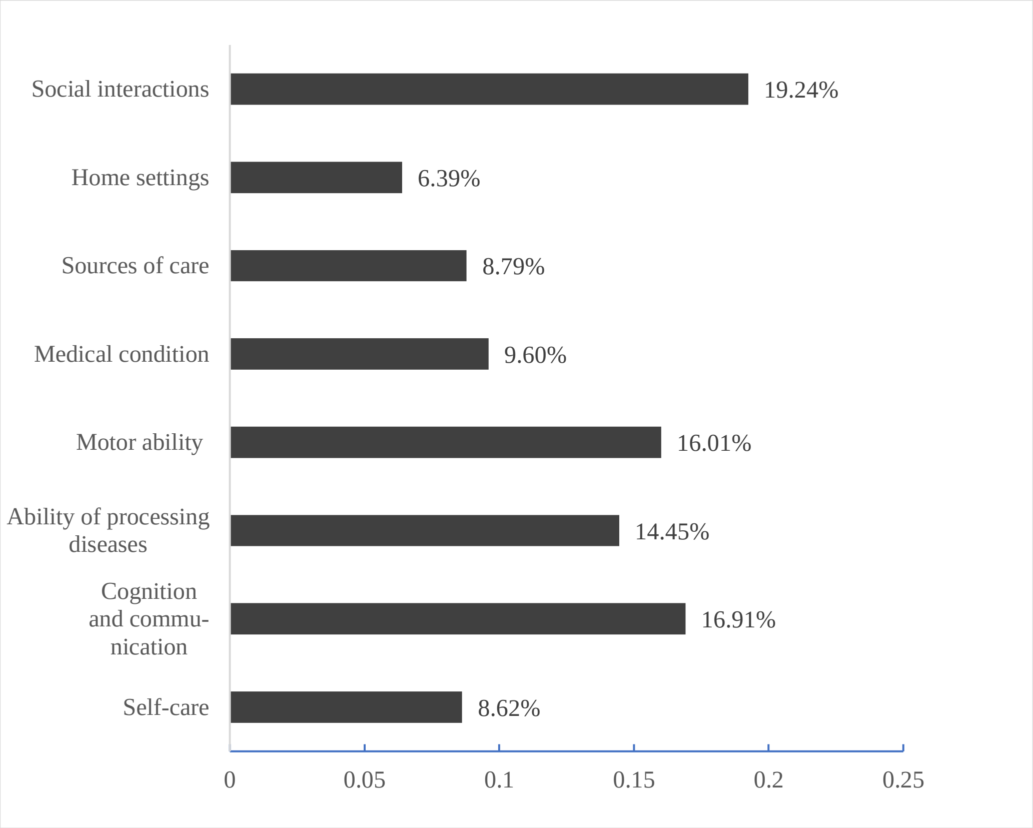

(1) k=1

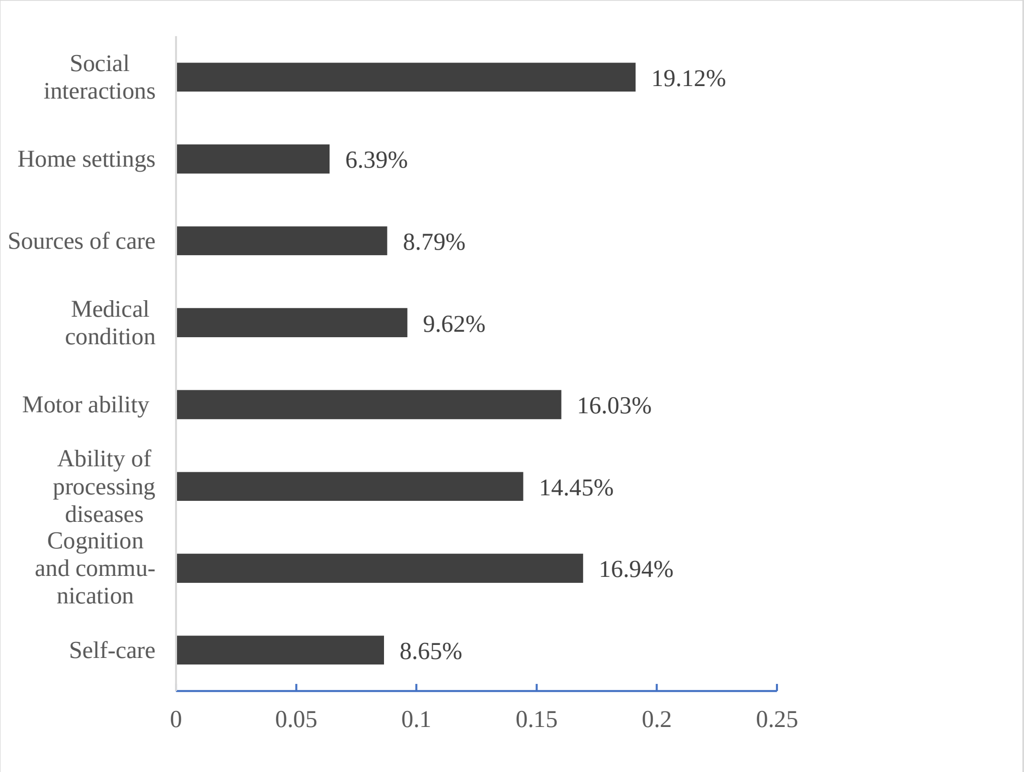

(2) k=2

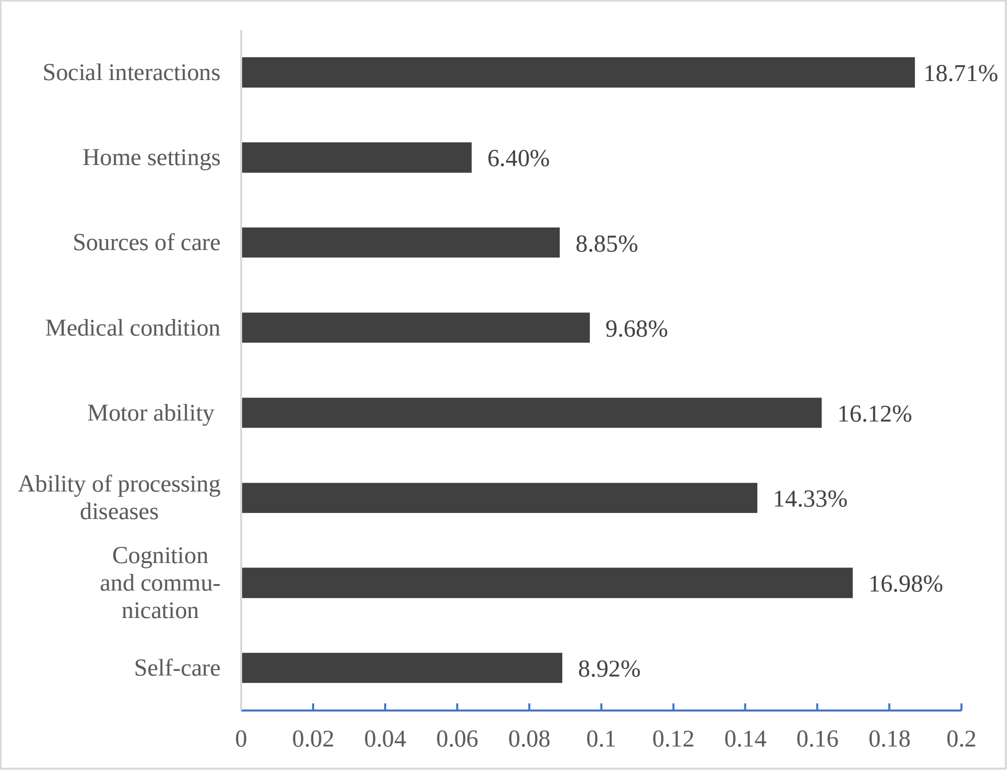
Social interactions
Home settings
Sources of care
Medical condition
Motor ability
Ability of processing diseases
Cognition and commu-nication
Self-care
18.71%
6.40%
8.85%
9.68%
16.12%
14.33%
16.98%
8.92%
0
0.02
0.04
0.06
0.08
0.1
0.12
0.14
0.16
0.18
0.2

(3) k=3

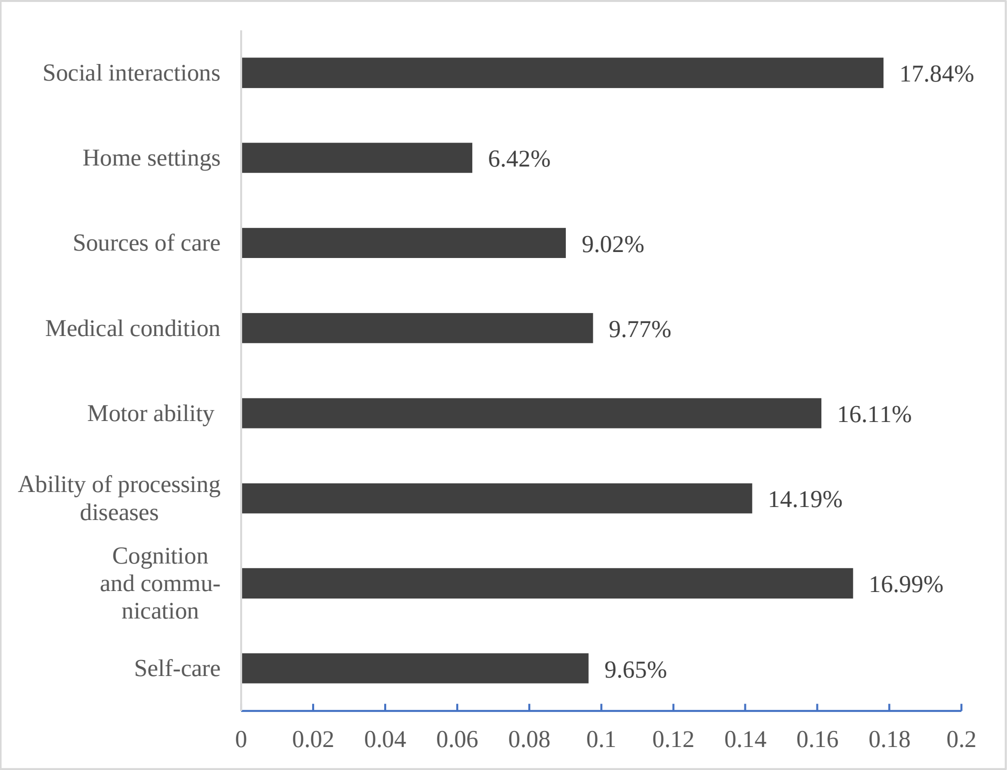
Social interactions
Home settings
Sources of care
Medical condition
Motor ability
Ability of processing diseases
Cognition and commu-nication
Self-care
17.84%
6.42%
9.02%
9.77%
16.11%
14.19%
16.99%
9.65%
0
0.02
0.04
0.06
0.08
0.1
0.12
0.14
0.16
0.18
0.2

(4) k=4

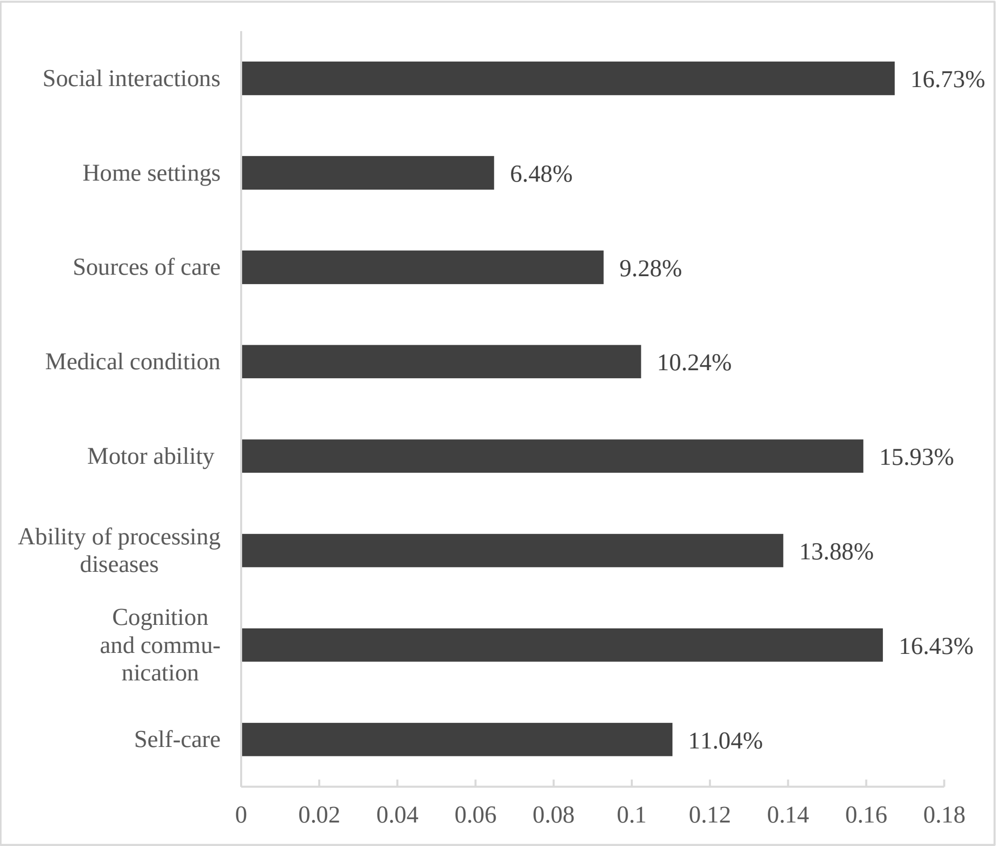
Social interactions
16.73%
Home settings
6.48%
Sources of care
9.28%
Medical condition
10.24%
Motor ability
15.93%
Ability of processing diseases
13.88%
Cognition and commu-nication
16.43%
Self-care
11.04%
0
0.02
0.04
0.06
0.08
0.1
0.12
0.14
0.16
0.18

(5) k=5

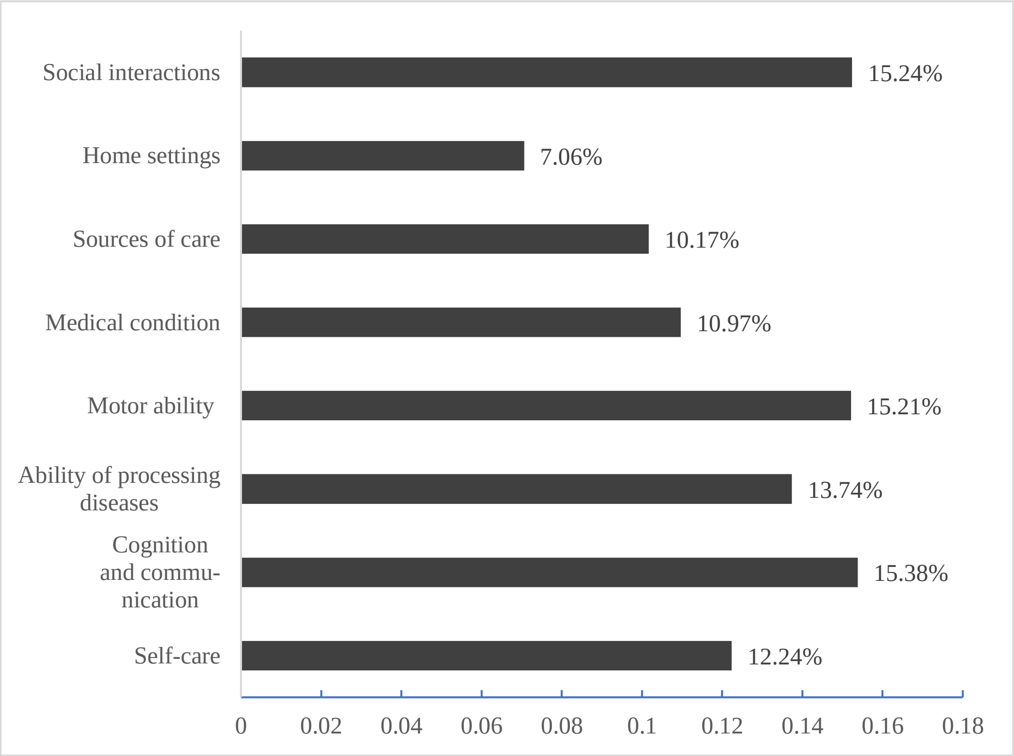
Social interactions
15.24%
Home settings
7.06%
Sources of care
10.17%
Medical condition
10.97%
Motor ability
15.21%
Ability of processing diseases
13.74%
Cognition and commu-nication
15.38%
Self-care
12.24%
0
0.02
0.04
0.06
0.08
0.1
0.12
0.14
0.16
0.18

(6) k=6

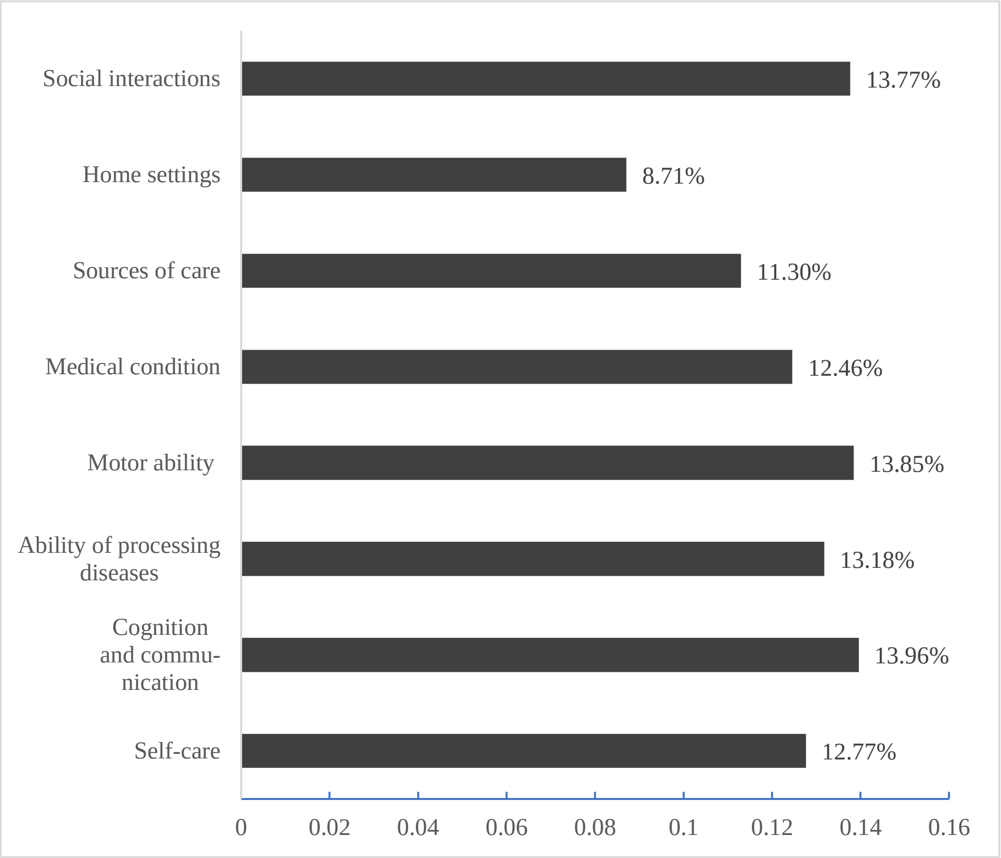
Social interactions
13.77%
Home settings
8.71%
Sources of care
11.30%
Medical condition
12.46%
Motor ability
13.85%
Ability of processing diseases
13.18%
Cognition and commu- nication
13.96%
Self-care
12.77%
0
0.02
0.04
0.06
0.08
0.1
0.12
0.14
0.16

(7) k=7

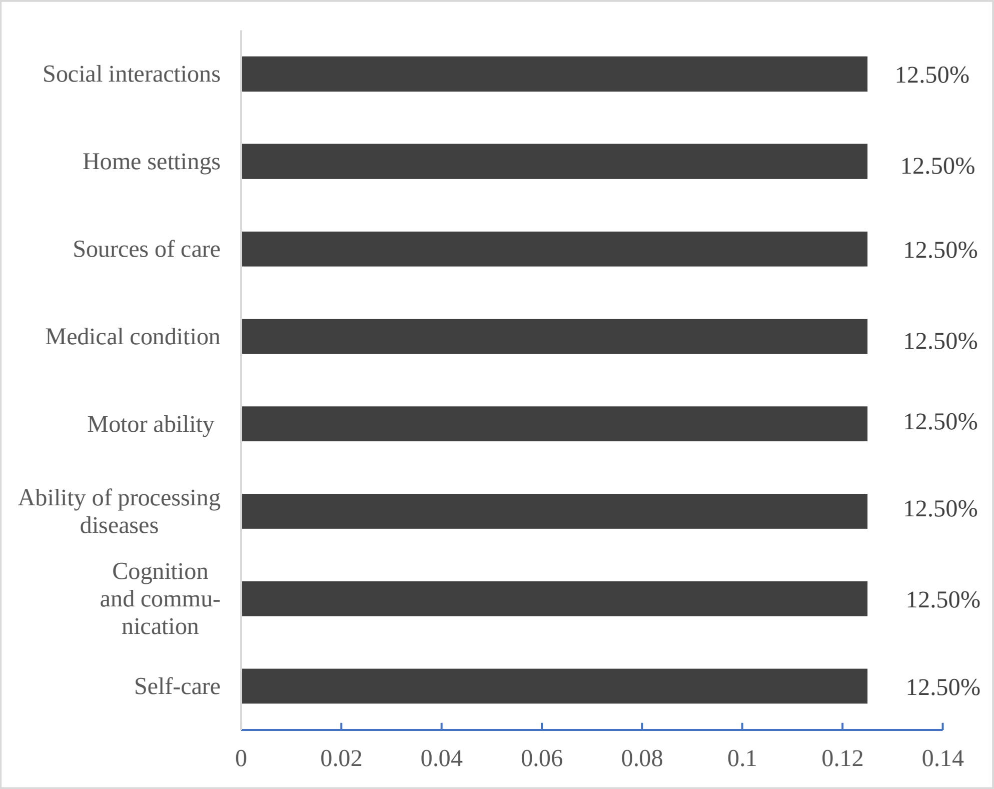
Social interactions
12.50%
Home settings
12.50%
Sources of care
12.50%
Medical condition
12.50%
Motor ability
12.50%
Ability of processing diseases
12.50%
Cognition and commu- nication
12.50%
Self-care
12.50%
0
0.02
0.04
0.06
0.08
0.1
0.12
0.14

(8) k=8

Figure S1. M_0 value under different dimensions

Note. K=the number of dimensions.

Table S1. Characteristics among different multidimensional deprivation methods

	**Con**	**Foc**	**Mon**	**SA**	**AA**	**UPDT**	**MPDT**	**SMD**
HDI	√	√	√	√	√	no	no	no
HPI	no	√	no	√ (if $\varepsilon=1$)	√ (if $\varepsilon=1$)	no	no	no
UH	no	√	no	√	√	no	no	no
IH	no	√	no	√	no	no	no	√
AF	no	√	√ (if $\alpha>0$)	√	no	no	no	no
MPI	no	√	no	√	no	no	no	no
MFGT	√	√	√	√	no	no	no	√
BC	√	√	√	√	√ (if $\alpha=\varepsilon$)	no	√	√

Note. **Con**: continuity; **Foc**: focus; **Mon**: monotonicity; **SA**: subgroup additivity; **AA**: attribute additivity; **UPDT**: unidimensional Pigou-Dalton transfer; **MPDT**: multidimensional Pigou-Dalton transfer; **SMD**: sensitivity to multiple deprivation; **HDI**: Human Development; **HPI**: Human Poverty; **UH**: Union Headcount; **IH**: Intersection Headcount; **AF**: Alkire-Foster; **MPI**: Multidimensional Poverty; **MFGT**: multiplicative FGT; **BC**=Bourguignon-Chakravarty.

Table S2. The selection and assigned values in 29 indicators

Dimensions-indicators	Assigned values of 29 indicators
**Self-care**	
ADL	This refers primarily to the ability to maintain basic life care, including the difficulty of bathing, eating, dressing, using the toilet, controlling urine and feces. The measurement of the difficulty degree of each indicator as follows: "with difficulty but able to complete", "with difficulty and need help", "unable to complete" =1; "Without any difficulty" =0.
IADL	It is the observation variable of the elderly's social activity ability, which consisted of housework, cooking, shopping and financial management, medicine. The measurement of the difficulty degree of each indicator as follows: "with difficulty but able to complete", "with difficulty and need help", "unable to complete" =1; "Without any difficulty" =0.
Frailty	Physical Frailty Phenotype was used to measure frailty, which consisted of weight loss, weakness, slowness, low energy expenditure, exhaustion. The score range of PFP scale is 0~5. When the score is 3 and higher, it is defined as frailty=1; otherwise=0.
**Motor ability**	
Balance	Stand with the heel side of one foot touching the big toe of the other for 10 seconds: "unable to complete" =1; "Without any difficulty" =0.
Falls	Have you fallen in the past two years: falls =1; non-falls =0.
Postural transition	Get up after sitting in your chair for a long time: "with difficulty but able to complete", "with difficulty and need help", "unable to complete" =1; "Without any difficulty" =0.
Arm stretching	Extend your arms up along your shoulders: "with difficulty but able to complete", "with difficulty and need help", "unable to complete" =1; "Without any difficulty" =0.
Mobility	Climb several flights of stairs without stopping: "with difficulty but able to complete", "with difficulty and need help", "unable to complete" =1; "Without any difficulty" =0.
**Ability of processing diseases**	
Receiving medical services	In the past month, have you visited a medical institution or received in-patient medical care: have been to=1; not have been to=0.
Exceptional treatment	Surgical infusion and acupuncture for any treatment: have been to=1; not have been to=0.

Self-treatment	In the past month, have you self-treated yourself in any of the following ways (note: prescriptions are not included here)? "buy over-the-counter or prescription drugs", "use traditional Chinese herbal medicine", "take health care products such as vitamins", "use health care equipment" =0; "none" =1.
Assistive Device	Do you use the following AIDS: "crutches", "walkers", "manual wheelchairs", "electric wheelchairs" =1; none of them=0.
**Cognitive mental status and communication**	
Cognition	The cognitive function of the elderly is usually measured by three aspects of mental state episodic memory ability and visual spatial ability. CHARLS project team asked interviewees to answer five mathematical calculation questions whether they knew the date of the year or month at the time of interview. One point was scored for each correct answer to the questions of the week and season, and the total score was the mental state score (range: 0-10 points). By asking respondents 10 words and asking them to recall the words at two different times, 0.5 points were scored for each pair of words recalled. The mean value of the two recall scores was the score of episodic memory ability (range :0~10 points). Visual spatial ability measured by pressing the figure painting, painting for 1 point, otherwise don't score (range: 0 ~ 1). Cognitive function in the elderly by three aspects including mental state, Episodic memory ability and visual spatial ability scored (range: 0 ~ 21 points). When the score was equal to or higher than the average (9.7 points), participants had higher cognitive functions, otherwise had low cognitive function. In this paper, below 9.7 points=1; higher than 9.7=0.
Memory	How do you feel about your memory now: "excellent", "very good", "good", "general" =0; "Poor" =1.
Vision	Including near object vision and distant vision: "Excellent", "very good", "good", "so-so" =0; "bad" =1.
Hearing	How do you feel about your hearing: "Excellent", "very good", "good", "so-so" =0; "bad" =1.
Sadness	To feel sad, depressed, or depressed: "No", "mild", "moderate" =0; "serious", "very serious" =1.
Depression	The Center for Epidemiologic Studies Depression Scale (CES-D), was used in this paper. CES-D consists of 10 items, in which respondents are asked about their feelings and behaviors in the past week, and are given 0~3 points according to the depression degree reflected in the items from low to high, with a total score of 0~30 points. The higher the score is, the more serious the depression is, and generally 10 points or above are considered as having depressive symptoms. a score of 0~9 was defined as no depressive symptoms=0;

	a score of 10~30 was defined as having depressive symptoms=1.
**Medical condition**	
Chronic disease	Have you been diagnosed with [conditions listed below, read one by one] by a doctor: hypertension, Diabetes, Dyslipidemia, Cancer or malignant tumor, Chronic lung diseases, Liver disease, Heart attack, Stroke, Kidney disease, Stomach or other digestive disease, Emotional, nervous, or psychiatric problems, Memory-related disease, Arthritis or rheumatism, Asthma =1; none=0.
Physical disabilities	Whether you have a physical disability: yes=1; none=0.
**Sources of care**	
Access of caregiver	If you need to be taken care of in daily life, such as eating, dressing, you will have a family member (other than your spouse) or a friend who can take care of you in the long term: no=1; yes=0.
Living arrangement	Living arrangement: “live with their children in a same house”, “in a same dwelling or courtyard” =0; “in a same village”, “in another household in this city” “in another province” =1.
**Home settings**	
Elevator in a four-story apartment	Housing types: without elevator=1; with elevator=0.
Accessibility	Do you have wheelchair accessible around your place: no=1; yes=0.
**Social interactions**	
Interacted with friends	Have you done any of this activity in the last month: no=1; yes=0.
Community club	Have you done any of this activity in the last month: no=1; yes=0.
Provide help to friends	Have you done any of this activity in the last month: no=1; yes=0.
Outdoor activities	Have you done any of this activity in the last month: no=1; yes=0.
Community-related organization	Have you done any of this activity in the last month: no=1; yes=0.
